# A new, unquenched intermediate of LHCII

**DOI:** 10.1016/j.jbc.2021.100322

**Published:** 2021-01-23

**Authors:** Fei Li, Cheng Liu, Simona Streckaite, Chunhong Yang, Pengqi Xu, Manuel J. Llansola-Portoles, Cristian Ilioaia, Andrew A. Pascal, Roberta Croce, Bruno Robert

**Affiliations:** 1 Key Laboratory of Plant Resources, Institute of Botany, Chinese Academy of Sciences, Beijing, China; 2 Université Paris-Saclay, CEA, CNRS, Institute for Integrative Biology of the Cell (I2BC), Gif-sur-Yvette, France; 3 Department of Physics and Astronomy, Faculty of Sciences, VU University Amsterdam, Amsterdam, The Netherlands

**Keywords:** light-harvesting complex, NPQ, photoprotection, resonance Raman, LHCII, α-DM, *n*-dodecyl-α-D-maltoside, β-DM, *n*-dodecyl-β-D-maltoside, α-DM-LHCII, LHCII purified with *n*-dodecyl-α-D-maltoside, β-DM-LHCII, LHCII purified with *n*-dodecyl-β-D-maltoside, Chl *a*, chlorophyll *a*, Chl *b*, chlorophyll *b*, LHC, light harvesting complex, LHCII, light harvesting complex II, NPQ, non-photochemical quenching, qE, energy dependent component of NPQ, Qy, light absorption due to electronic transition along the *y*-axis of the chlorophyll molecule

## Abstract

When plants are exposed to high-light conditions, the potentially harmful excess energy is dissipated as heat, a process called non-photochemical quenching. Efficient energy dissipation can also be induced in the major light-harvesting complex of photosystem II (LHCII) *in vitro*, by altering the structure and interactions of several bound cofactors. In both cases, the extent of quenching has been correlated with conformational changes (twisting) affecting two bound carotenoids, neoxanthin, and one of the two luteins (in site L1). This lutein is directly involved in the quenching process, whereas neoxanthin senses the overall change in state without playing a direct role in energy dissipation. Here we describe the isolation of an intermediate state of LHCII, using the detergent *n*-dodecyl-α-D-maltoside, which exhibits the twisting of neoxanthin (along with changes in chlorophyll–protein interactions), in the absence of the L1 change or corresponding quenching. We demonstrate that neoxanthin is actually a reporter of the LHCII environment—probably reflecting a large-scale conformational change in the protein—whereas the appearance of excitation energy quenching is concomitant with the configuration change of the L1 carotenoid only, reflecting changes on a smaller scale. This unquenched LHCII intermediate, described here for the first time, provides for a deeper understanding of the molecular mechanism of quenching.

During the first steps of the photosynthetic process, solar photons are absorbed by specialized light-harvesting complexes (LHCs), and the resulting excitation energy is transferred to reaction center pigments, where it is converted into a chemical potential. In low-light conditions, most of the photons absorbed lead to a charge separation event at the reaction center (1, 2). However, when the absorbed energy is in excess of that which can be used for energy transduction, the overaccumulation of excited states can result in damage to the photosynthetic membrane, in particular, *via* the production of reactive oxygen species. In high-light conditions, the antenna system of plants and algae reorganizes reversibly, creating energy traps that dissipate the excess excitation as heat (3, 4, 5). This regulatory mechanism is known as non-photochemical quenching of chlorophyll fluorescence (NPQ). NPQ is a multi-component phenomenon whose fastest phase, qE (energy-dependent quenching), is triggered by the ΔpH across the thylakoid membrane, itself resulting from photosynthetic activity. qE induction and relaxation occurs in seconds, and it cannot involve *de novo* protein synthesis but rather corresponds to a reorganization of the existing photosynthetic membrane.

Over the last two decades, a large number of studies have been performed to gain insight into the molecular mechanisms underlying qE (6, 7, 8, 9, 10, 11). Proteins of the LHC family have the in-built capacity to quench excitation energy (6, 12, 13, 14, 15). LHCII, the major antenna complex in higher plants, occurs as a trimer of nearly identical monomers. Each monomer binds 14 chlorophyll molecules (8 Chl *a* and 6 Chl *b*), three tightly-bound carotenoids (two luteins and one 9-*cis* neoxanthin), and one weakly-bound violaxanthin carotenoid (16). Self-association of this protein upon detergent removal causes a significant quenching of excitation energy, first reported some four decades ago (17). Femtosecond transient absorption measurements conducted on LHCII aggregates showed that excitation quenching occurs through energy transfer from Chl *a* to the S_1_ excited state of a lutein molecule (6), more precisely the LHCII-bound lutein absorbing at 495 nm, bound to the protein site termed L1 (18, 19). The short lifetime of the carotenoid S_1_ excited state ensures an efficient dissipation of the excitation energy as heat (6, 20). More recently a similar quenching mechanism was observed in several isolated high-light-inducible proteins (21), members of the LHC superfamily that are in a permanently-quenched state and that are believed to be the ancestors of LHC antenna proteins (22, 23). Excitation energy transfers from Chls to different carotenoid states have been observed in quenched monomeric LHCII (10), quenched LHCII trimers (24, 25), the minor antenna CP29 (15), and in thylakoid membranes exposed to high light (26).

The formation of the quenching site in LHCII involves structural changes in a number of bound cofactors, revealed by resonance Raman spectroscopy (12). Changes in the interaction state of several chlorophylls with the protein host were observed, as well as in the configuration of the neoxanthin molecule (27), and more recently, in that of L1 lutein (14), consistent with the role of L1 as the quenching species. The amplitude of the neoxanthin change is strictly correlated with the extent of quenching in LHCII as well as in intact chloroplasts and leaves (6), and this carotenoid thus appears as a reporter of structural changes leading to quenching, a proposition that has recently been supported by molecular dynamics simulations (28). LHC proteins were proposed to be the major site of quenching in plants, which would occur through a subtle equilibrium between two LHC conformations (29, 30).

The LHCII structure is very sensitive to its environment (12, 24, 25, 27, 28, 31, 32). LHCII purified in the presence of either α- or β-dodecyl-D-maltoside (α-DM or β-DM) displays slightly different electronic properties (31, 33), and the H-bonding interactions of two bound chlorophyll *a* molecules are sensitive to the detergent used (34). In this work, we have studied the vibrational properties of the different pigments bound to LHCII in the presence of α-DM or β-DM. We show that the Raman signals of Chls *a* and *b* and neoxanthin that were previously associated with LHCII quenching are already present in LHCII purified in the presence of α-DM—even though this preparation is unquenched—while aggregation-induced quenching affects the L1 carotenoid-binding site alone. The molecular structure of Chl *a*, Chl *b,* lutein and neoxanthin pigments and those of the detergents used for the purification of LHCII are dispayed in Figure 1.

**Figure 1:**
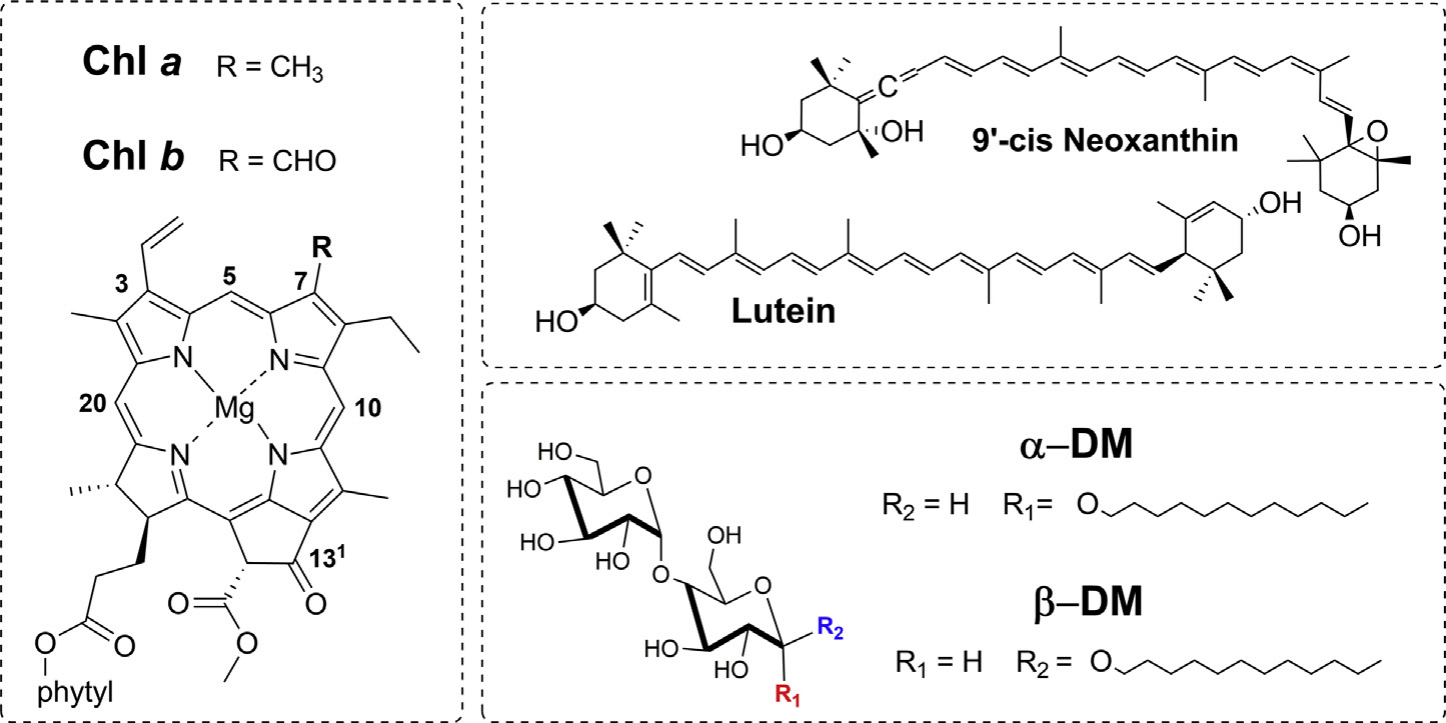
**Molecular structures of the LHCII tightly bound pigments and the detergents used for LHCII purification.** Molecular structures of chlorophyll *a*, chlorophyll *b*, 9′-cis neoxanthin, *all-trans* lutein, α-dodecyl-D-maltoside (α-DM), and β-dodecyl-D-maltoside (β-DM).

## Results

### Influence of the stereochemistry of dodecyl-maltoside on LHCII electronic properties

**Figure 2:**
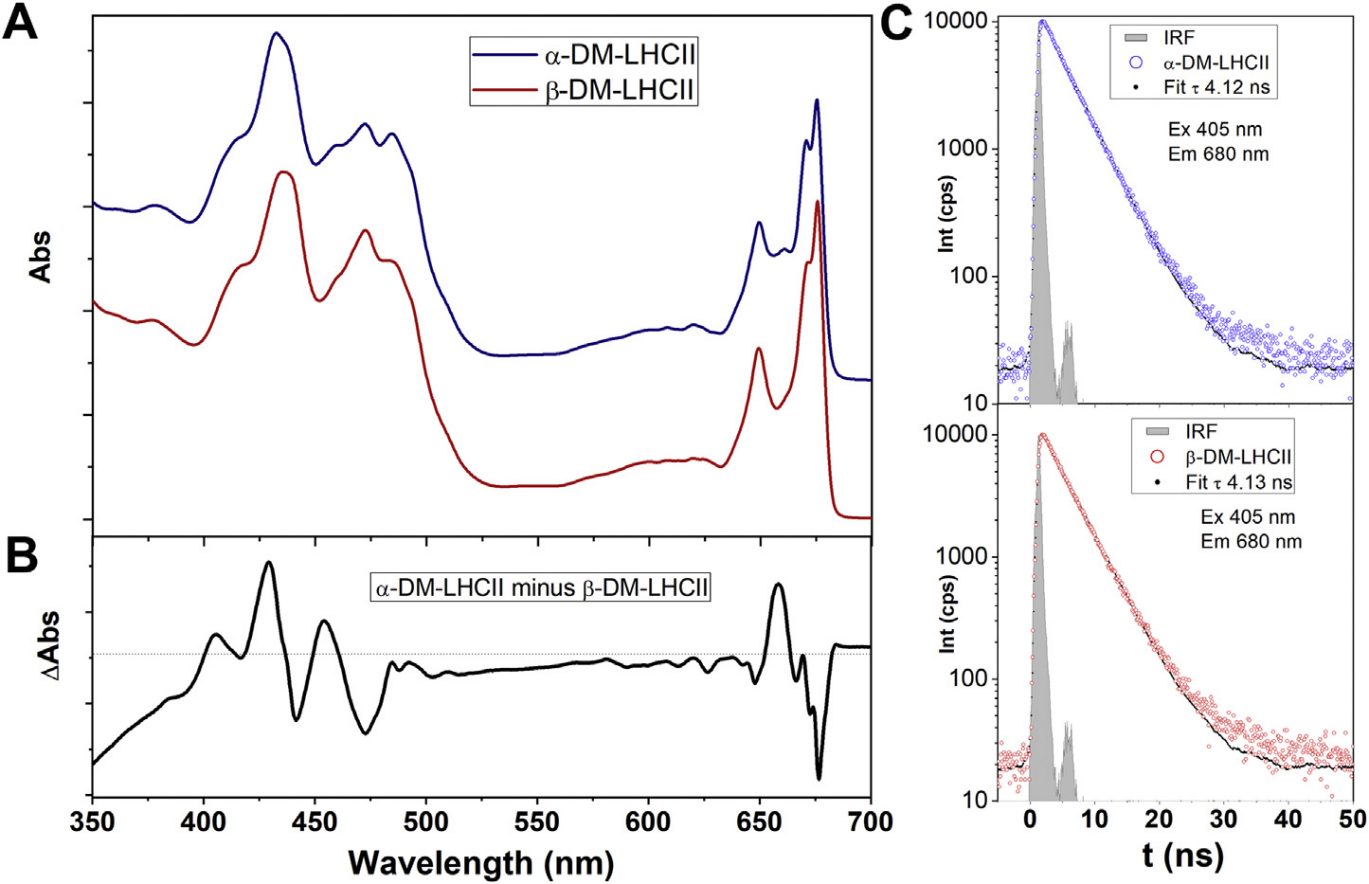
**Absorption and time-resolved fluorescence spectra of LHCII.***A*, absorption spectra at 4.2 K of LHCII in α-DM and β-DM (*blue* and *red*, respectively). *B*, difference spectrum “α-DM-LHCII minus β-DM-LHCII.” *C*, time-resolved fluorescence of α-DM-LHCII (*blue*) and β-DM-LHCII (*red*), excited at 405 nm with emission recorded at 680 nm. The *grayed region* represents instrumental response function in time domain.

As already reported (31, 33, 34), both the carotenoid and chlorophyll absorption regions of LHCII exhibit differences between the α-DM-solubilized *versus* the β-DM-solubilized protein (Fig. 2*A*). In the blue region, the Soret absorption transition of Chl is perturbed—in α-DM, an increase in intensity is seen at 432 nm, concomitant with a loss at 438 nm. The carotenoid contributions appear to lose intensity at 480 and 472 nm and gain intensity at 457 nm. In the Chl Q_y_ region, a new band is present at 660 nm in LHCII isolated in α-DM, while the intensity of the transitions at 676 and 672 nm is reduced. All these changes are more easily observed in the difference spectrum (Fig. 2*B*). None of these changes in absorption induces any observable differences in fluorescence properties (*e.g.*, Fig. 2*C*).

### Influence of DM stereochemistry on LHCII chlorophylls

Resonance Raman spectroscopy has seen extensive application to the assessment of pigment structure and interactions in photosynthetic proteins (35, 36, 37) and played a vital role in revealing the modifications to cofactor structure associated with the LHCII “conformational change” model of NPQ (12, 27). The Raman spectra of chlorophyll molecules are particularly rich, containing a number of bands that are sensitive to the chlorophyll conformation and to its interactions with the immediate environment. Upon aggregation-induced quenching in LHCII, two bound Chl *a* molecules lose a hydrogen bond to their conjugated keto carbonyl group on position C13^1^, while one or two Chls *b* gain H-bonds to their conjugated formyl group at position C7 (observed for excitations at 413.1 and 441.6 nm, respectively [27]).

**Figure 3:**
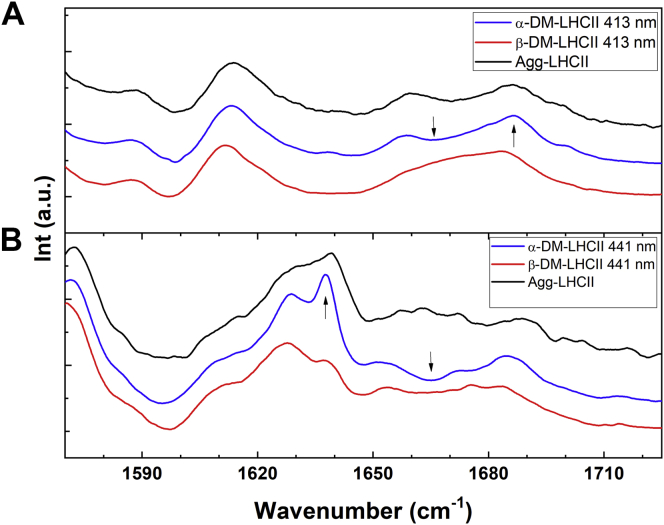
**High-frequency region of resonance Raman spectra of LHCII**. Resonance Raman spectra at 77 K in the 1540 to 1720 cm^−1^ region for α-DM-LHCII (*blue*), β-DM-LHCII (*red*), and LHCII aggregates (*black*) excited at (*A*) 413.1 and (*B*) 441.6 nm.

Chlorophyll resonance Raman spectra of the two LHCII preparations at 77 K are presented in Figure 3. Comparing α-DM-LHCII relative to β-DM-LHCII for the Chl *a* excitation at 413.1 nm (Fig. 3*A*), there is a clear increase in contributions on the high-frequency side of the envelope of bands in the 1660 to 1700 cm^−1^ region, which corresponds to stretching modes of Chl *a* keto groups conjugated with the macrocycle. This increase around 1690 cm^−1^ is accompanied by a corresponding decrease at lower frequency at ∼1670 cm^−1^ (shown by black arrow heads in Fig. 3*A*). As discussed elsewhere (34), this reflects the loss of an H-bond to probably two LHCII-bound Chl *a* molecules, at the level of their conjugated keto carbonyl group.

Excitation at 441.6 nm yields spectra in which Chl *b* vibrational modes dominate. The high-frequency region thus corresponds to stretching modes of both conjugated carbonyl groups of Chl *b* – C7-formyl and C13^1^-keto, in the 1620 to 1660 and 1660 to 1700 cm^−1^ ranges, respectively. When the two preparations are compared at this wavelength (Fig. 3*B*), an intense contribution is observed for α-DM-LHCII at 1637 cm^−1^ that is not present in β-DM-LHCII, accompanied by a decrease in the intensity of the contribution at 1666 cm^−1^ (shown by black arrow heads in Fig. 3*B*). This indicates that the formyl carbonyl of at least one Chl *b*, which is free from interactions in β-DM-LHCII, becomes strongly H-bonded in α-DM-LHCII. It is particularly interesting to note that the changes in Chl *a* and *b* interactions observed for α-DM-LHCII are strikingly similar to those seen upon aggregation-induced quenching in LHCII (27) (see spectra of aggregates in Fig. 3).

### Influence of DM stereochemistry on LHCII-bound neoxanthin

Carotenoid resonance Raman spectra are mainly composed of four groups of bands, termed ν_1_–ν_4_. The ν_1_ band above 1500 cm^−1^ arises from stretching vibrations of C=C double bonds, and its frequency depends on the length of the π-electron conjugated chain and on the molecular configuration of the carotenoid (38, 39, 40, 41, 42, 43). The ν_2_ band at 1160 cm^−1^ contains contributions from stretching vibrations of C–C single bonds, coupled with C–H in-plane bending modes. This ν_2_ region is a fingerprint for the assignment of carotenoid isomerization states (40, 44). At 1000 cm^−1^, the ν_3_ band arises from in-plane rocking vibrations of the methyl groups attached to the conjugated chain, coupled with in-plane bending modes of the adjacent C–H's (38). It was recently shown to be a fingerprint of the conjugated end-cycle configuration (45, 46) and sensitive to the presence of a conjugated allene group (47, 48). Finally, the ν_4_ band, around 960 cm^−1^, arises from C–H out-of-plane wagging motions coupled with C=C torsional modes (38). When the carotenoid conjugated system is planar, these out-of-plane modes are not coupled with the electronic transition and thus exhibit little intensity. However, distortions around C–C single bonds increase the coupling of (some of) these modes with the electronic transition, resulting in an increase in their intensity (49).

**Figure 4:**
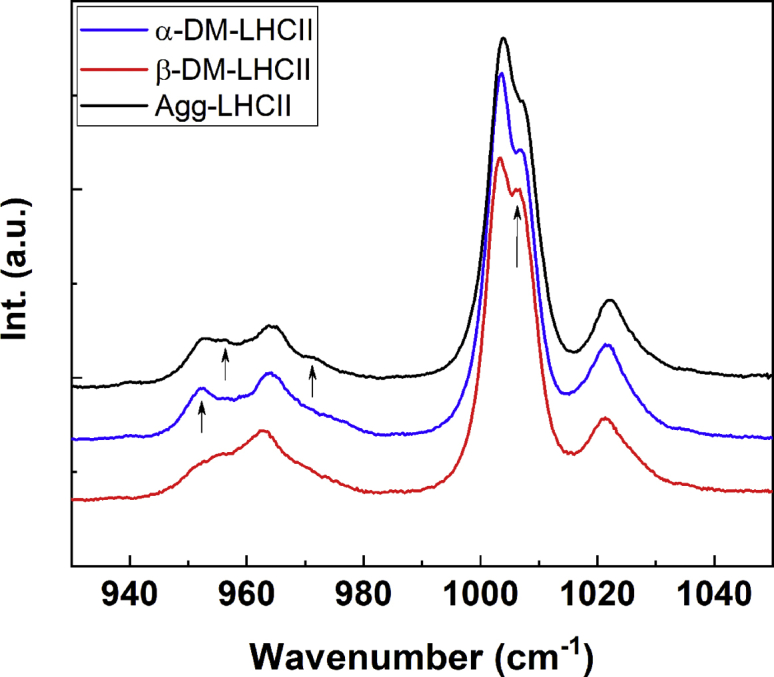
**ν_3_and ν_4_regions of resonance Raman spectra of LHCII.** Resonance Raman spectra at 77 K in the 930 to 1050 cm^−1^ region for α-DM-LHCII (*blue*), β-DM-LHCII (*red*), and LHCII aggregates (*black*) excited at 488.0 nm.

Excitation of LHCII at 488.0 nm yields Raman spectra in which contributions of the bound neoxanthin molecule dominate (19). The spectra of α-DM- and β-DM-LHCII excited at 488.0 nm at 77 K do not exhibit substantial differences in their ν_1_ and ν_2_ regions (data not shown). Hence, this indicates that the conjugation length of Neo and its configuration (*cis*) are not altered by purification with different detergents. However, significant differences are observed in the ν_3_ and ν_4_ bands (Fig. 4). In the ν_4_ region, both samples display a band at 963 cm^−1^, whereas an additional component at 952 cm^−1^ gains intensity for α-DM-LHCI, indicating that neoxanthin undergoes a twist in the presence of this detergent, which is not present in β-DM-LHCII. This is accompanied by a change in the structure of the ν_3_ band. In the latter region, α-DM- and β-DM-LHCII both show a doublet characteristic of allene-containing carotenoids. However, the relative intensity of the two components at 1003.3 and 1006.6 cm^−1^ is different, consistent with a structural change of the neoxanthin between the two samples. Once again, the changes observed for α-DM-LHCII are similar to those seen in the quenched state of LHCII (see spectrum of LHCII aggregates in Fig. 4). However, the spectrum for aggregated LHCII displays additional components at 956 and 971 cm^−1^, which are absent in the spectrum of LHCII in α-DM. Note that LHCII aggregation produces identical spectra, whether the starting point is α-DM- or β-DM-LHCII. These specific features at 956 and 971 cm^−1^ have previously been assigned to a distortion of the lutein1 carotenoid (14), the pigment identified as the quenching species in aggregated LHCII (6).

### Structure of the lutein1 carotenoid

**Figure 5:**
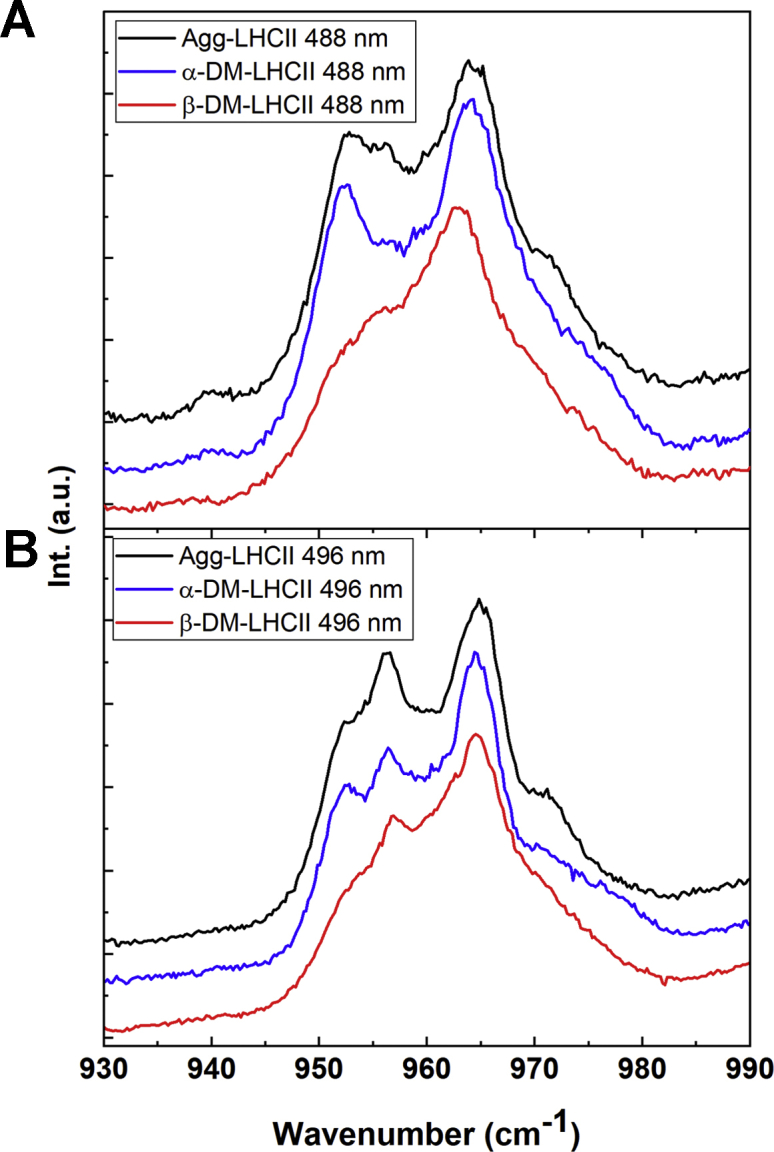
**ν_3_region of LHCII resonance Raman spectra of LHCII.** Resonance Raman spectra at 77 K in the ν_4_ region for LHCII in α-DM (*red*), β-DM (*blue*), and in the aggregated form (*black*), for excitation at 488.0 (*A*) and 496.5 nm (*B*).

As discussed, the resonance Raman spectra obtained for LHCII when solubilized in α-DM or in the aggregated form display no significant differences at 413.1 and 441.6 nm, where Chl *a* and *b* molecules contribute, respectively. Excitation at 488.0 nm generates very similar spectra between these two samples, but with small differences in the ν_4_ region. A zoom of this region is presented in Figure 5, for excitation at 488.0 and 496.5 nm (dominated by neoxanthin and lutein1, respectively) for LHCII in β-DM, in α-DM, and upon aggregation by detergent removal. The spectra of α-DM-LHCII are globally similar to the quenched aggregates, reflecting the major twisting of the neoxanthin carotenoid, whereas aggregated LHCII exhibits additional components at 956 and 971 cm^−1^, which are absent for α-DM-LHCII. These bands are more clearly seen for excitation at 496.5 nm, corresponding to the absorption peak of lutein1 (18, 19).

These differences between α-DM-LHCII and the quenched aggregates are strikingly similar to those observed upon aggregation of LHCII purified from the *npq2* mutant, which lacks neoxanthin. In the latter case, the spectral changes were attributed to a change in conformation of the lutein1 carotenoid, which in the WT complex is considerably masked by the larger changes in neoxanthin signal (14).

## Discussion

Changes in pigment conformation in the major plant antenna protein LHCII, associated with the appearance of fluorescence quenching, have been documented for some 25 years (27). Removal of the solubilizing detergent in β-DM-purified LHCII leads to a tenfold or more decrease in its fluorescence level, along with structural changes in the binding pockets of two Chls *a*, one or two Chls *b*, and the neoxanthin and lutein1 carotenoids. Here we show that LHCII purified using the stereoisomer α-DM exhibits intermediate properties, where all of the quenching-associated changes are already present apart from that in L1, even though this LHCII preparation is unquenched (see fluorescence lifetimes in Fig 2*C*). Aggregation of α-DM-LHCII induces quenching, with the change in the L1 carotenoid now present. Note that the presence of additional LHCII conformations (*i.e.*, more than two) has already been inferred from single-molecule and time-resolved fluorescence experiments on LHCII, as well as molecular dynamics simulations (13, 29).

All these pigment structural changes were up to now directly associated with the appearance of quenching. The ability of the detergent α-DM to stabilize an unquenched intermediate, which has not been observed previously, allows us to disentangle the molecular changes leading to quenching in LHCII. Indeed, the only change directly associated with quenching is that occurring in the L1 binding pocket. A careful analysis of the 488.0-nm resonance Raman spectra obtained previously for quenched LHCII, whether in aggregates, crystals, or gels (6, 12, 14, 27), reveals that all the samples display the additional components at 956 and 971 cm^−1^ in the ν_4_ region. This indicates that, in each case, the change in L1 configuration associated with quenching has indeed occurred. Thus the configuration of the L1 carotenoid is intrinsically linked with the quenching process, consistent with its attribution as the site of quenching (6, 15).

The significance of the changes affecting Chls *a* and *b* and neoxanthin, upon aggregation of β-DM-LHCII, may thus be questioned. It could be argued that β-DM stabilizes LHCII in a non-native state, which relaxes upon detergent removal, and that α-DM-LHCII and the aggregates represent the only two states observed *in vivo* (in unquenched and quenched conditions, respectively). However, it was shown that the changes occurring upon qE induction in chloroplasts and leaves do indeed involve twisting of the bound neoxanthin, and this twisting signal is proportional to the extent of quenching (6), just as for aggregation of β-DM-LHCII (and not of α-DM-LHCII). This rather suggests a two-step mechanism for qE: a state similar to β-DM-LHCII is the major light-harvesting state, and this converts to the quenched (aggregate-like) state *via* an unquenched intermediate similar to α-DM-LHCII. The fact that the “α-DM effect” has not been observed before tends to suggest that this intermediate is at least relatively rare. However, given the complexity of these kinds of measurements on intact chloroplasts and leaves, and the small sizes of the changes involved, they have generally been performed in fully-unquenched and fully-quenched states, and not so much at intermediate levels of quenching. It is therefore possible that conditions do exist where a structure similar to α-DM-LHCII is present *in vivo* to a more significant extent. It could also be hypothesized that this intermediate state is involved in an as-yet-unknown aspect of qE or its regulation and/or in a different regulatory process requiring tuning of the pigment structure of LHCII. It is worth noting, in this regard, that qE *in vivo* is tightly regulated, and in a somewhat complex manner (*e.g.*, zeaxanthin, produced during exposure of leaves to high light, accelerates the onset of qE upon a second illumination (50)), whereas other (non-qE) phases of NPQ may also involve LHCII quenching (51). Further investigations along these lines should lead to a more profound understanding of the remarkable flexibility exhibited by the LHCII protein.

## Experimental procedures

α-DM- and β-DM-LHCII were isolated from *Arabidopsis thaliana* plants as described (52, 53). Thylakoid membranes at a Chl concentration of 1 mg/ml were solubilized by adding the same volume of buffer containing 1.2% α-DM or 2% β-DM, respectively. The mixture was gently vortexed for a few seconds, and unsolubilized material was removed by centrifugation at 17,000*g* for 10 min. The supernatant was then loaded onto a 0 to 0.1 M sucrose density gradient containing 10 mM Hepes pH 7.5, with 0.03% α-DM or 0.06% β-DM, respectively. The LHCII band was collected after overnight ultracentrifugation at 280,000*g*.

Quenched LHCII was prepared by detergent removal using SM-2 bioabsorbent beads (Bio-Rad), allowing for a tenfold reduction in fluorescence yield as determined by a mini PAM-I fluorimeter (Heinz Walz).

Time-resolved photoluminescence decay curves were acquired on an EI fluorescence plate reader (Edinburgh Instruments) using 4000 detection bins of 2 μs integration time. Excitation was with an Edinburgh EPL 405-nm picosecond diode laser, with a repetition rate of 5 MHz, and a 716/40 bandpass filter was placed between the sample and the detection system. All samples were measured in black 96-well plates with an optimal working volume of 150 μl. photoluminescence decay curves were mathematically fitted using FAST software (Edinburgh Instruments).

UV-visible absorption spectra were measured using a Varian Cary E5 double-beam scanning spectrophotometer, with a 1.0-cm pathlength cuvette. Samples were maintained at low temperature in a helium bath cryostat (Maico Metriks); 60% glycerol (v/v) was added to the sample to prevent devitrification.

Resonance Raman spectra at 77 K were obtained in a liquid nitrogen flow cryostat (Air Liquide), using a Jobin-Yvon U1000 Raman spectrophotometer equipped with a liquid-nitrogen-cooled, charge-coupled-device detector (Spectrum One, Jobin-Yvon). Laser excitations at 488.0, 496.5, and 501.7 nm, and 413.1 nm, were obtained with Coherent argon (Sabre) and krypton (Innova 90) lasers, respectively. Excitation at 441.6 nm was obtained with a Liconix helium–cadmium laser.

## Data availability

All data are contained within the article. Additional raw data are available upon request to the corresponding author.

## Conflict of interest

The authors declare that they have no conflicts of interest with the contents of this article.
